# Does Glycerin Used in *Varroa* Treatments Alter Propolis Quality?

**DOI:** 10.3390/insects16090871

**Published:** 2025-08-22

**Authors:** Freideriki Papakosta, Konstantia Graikou, Leonidas Charistos, Antigoni Cheilari, Fani Hatjina, Ioanna Chinou

**Affiliations:** 1 Laboratory of Pharmacognosy and Chemistry of Natural Products, Faculty of Pharmacy, National and Kapodistrian University of Athens, Panepistimiopolis, Zografou, 15771 Athens, Greece; freiderikipa@pharm.uoa.gr (F.P.); kgraikou@pharm.uoa.gr (K.G.); cheilarianti@pharm.uoa.gr (A.C.); 2 Department of Apiculture, Hellenic Agricultural Organisation ‘DIMITRA’, 63200 Nea Moudania, Greece; leocharistos@elgo.gr (L.C.); fhatjina@gmail.com (F.H.)

**Keywords:** *Varroa destructor*, propolis, GC-MS, glycerol residue, beekeeping treatments, sublimation, oxalic acid, formic acid, amitraz

## Abstract

In the current study, the impact of different acaricide treatments against *Varroa* (such as amitraz strips, oxalic and formic acid strips impregnated with glycerin, or the sublimation or instillation of oxalic acid) on glycerol residue levels of propolis was investigated. Propolis samples were collected from five beehives located in Chalkidiki (northern Greece), where all mentioned treatments were used and chemically analyzed and compared to control ones. Propolis samples were collected on days 7, 21, and 65 after the application of *Varroa* treatments, extracted with ethanol/water (70:30), silylated, and then analyzed using GC-MS, showing the predominance of diterpenes. The sublimation of oxalic acid and the amitraz treatment yielded a low glycerol residue (5.12% and 5.09% from 9.98% and 9.19%, respectively) in propolis specimens, while glycerin-impregnated oxalic acid strips led to elevated glycerol percentages (24.30% from 20.51%), unlike the reduced glycerol residues for all other treatments (instillation: 12.60% from 14.48% and glycerin-impregnated formic acid strips: 8.91% from 9.25%) and controls (3.27% from 6.30%). Furthermore, Principal Component Analysis (PCA) and the corresponding biplot illustrated how the sample composition varied across treatments and sampling days, highlighting the chemical constituent categories that most strongly contributed to these distinctions. These findings suggest that the use of glycerol-impregnated strips should be avoided in future beekeeping treatments against varroosis, as they could have a negative impact on the quality of propolis either for nutritional or medicinal applications.

## Simple Summary

The ectoparasitic honey bee mite *Varroa* destructor is among the greatest threats to apiculture. Beekeepers in Greece use various acaricide treatments like amitraz strips, oxalic or formic acid strips impregnated with glycerin, and the sublimation or instillation of oxalic acid to control *Varroa* mites. However, there is no information about the effect of the residues of those treatments on the quality of propolis. This study investigated the impact of each treatment on the chemical composition of propolis by periodically sampling it over a 65-day period and quantifying the potential percentage of glycerol residue. The results showed that the use of oxalic acid strips impregnated with glycerin deteriorated the quality of propolis, resulting in high percentages of glycerol residues over long periods, while in all the other beekeeping treatments, the glycerol residue was variably lower than varying percentages. PCA offered an overview of sample grouping based on the impact of treatments and their chemical composition. Overall, the findings indicate that out of the tested treatments, the most effective for controlling varroosis while ensuring the quality of propolis is the sublimation of oxalic acid. The use of glycerol-impregnated strips should be avoided in future treatments in order to preserve the quality of propolis.

## 1. Introduction

Propolis is a natural bee product that comprises many different chemical substances, such as waxes, resins, balsams, aromatic and ethereal oils, pollen, and other organic and inorganic molecules collected from the leaves, buds, and exudates of diverse trees and herbaceous plant species. It is a lipophilic, resinous, sticky hive product, which is used by bees for the protection of their hives either from intruders or from bacterial, viral, or fungal infections [1,2,3]. This defensive role of propolis is also verified from the name itself, which means “in front of the city” in Greek. The factors that have an influence on the complexity of the chemical composition of raw propolis are the season of collection as well as the botanical and geographical origin [3]. More than 800 constituents have been documented in propolis from the category of aliphatic acids and esters, aromatic acids and esters, flavonoids, phenolics, terpenoids, and others [4]. Regarding its pharmacological effects and health benefits, it has been used in traditional and alternative medicine for its anti-inflammatory, antioxidant, antibacterial, antiviral, antifungal, antiparasitic, and anti-allergic properties, as well as for its potential role against COVID-19 as a complementary treatment [5].

Propolis is collected by one of the most valuable insect species worldwide for agriculture, humans, and ecosystems: the western honey bee *Apis mellifera* L. Honeybees are indispensable as they sustain ecosystems through their role in pollination, with *Apis mellifera* being the most economically important pollinator worldwide [6]. For this reason, every factor that has a detrimental effect on bee health is a matter of universal concern and needs to be controlled. Specifically, one of the main causes of colony losses is an ectoparasitic mite named *Varroa destructor*. This parasite feeds on the body fat and hemolymph of bees in their mature and immature stages, contributing to the global decline in honey bee populations, and it is a vector for viruses, making it a major problem for apiculture [7]. For the control of *Varroa*, beekeepers use commercial chemicals that comprise synthetic acaricides such as fluvalinate, coumaphos, or amitraz, due to their effectiveness [8,9,10]. Specifically, amitraz is a formamidine derivative insecticide [N,N’-(methylimino-dimethylidyne) bis-2,4-xylidine (C_19_H_23_N_3_)] that can be applied via a sustained-release strip technique or through fumigation [11]. It is recommended to set two strips containing 500 mg of amitraz each per hive for about 6–8 weeks [12]. However, over the years, the overuse and misuse of these chemicals has resulted in pharmacological resistance from mites and the contamination of beehive products, respectively, threatening humans’ and honeybees’ health [13,14,15,16]. As a result of this fact, beekeepers are increasingly interested in using natural products and their derivatives, which are chemically complex and less likely to lead to mite resistance. Among these, the most commonly used, also approved as medicine by the European Medicines Agency (EMA), is oxalic acid, which, together with other organic acids such as formic acid, lactic acid, and plant essential oils (e.g., thymol), is commonly used [17,18]. These soft acaricides have low residue levels in bee products and can also be identified in honey naturally [17]. It has been documented in various studies that organic acids are preferably used when honey bee colonies are broodless [19,20], and in that case, the organic acids are applied through instillation or sublimation. However, another method widely used by beekeepers is by placing strips impregnated with the acaricide on the comb frames of a hive during the brood-rearing season. For the efficient distribution of these organic substances, several studies have suggested that glycerin could be added to the formulations as a solvent, as its viscosity helps to prolong the diffusion of the acid [21,22]. Although glycerin prolongs the presence of acaricide inside the beehive and aids in controlling mites, the chemical analysis of propolis by Pyrgioti et al. [1] showed high percentages of glycerol residue due to the use of glycerin-impregnated strips for anti-*Varroa* treatments. However, other bee products such as honey and wax seem, according to the literature, to not be affected by this technique [21].

The collection area of the assayed propolis samples was the Chalkidiki region (northern Greece), where the Department of Apiculture of the Institute of Animal Husbandry Science, ELGO-DIMITRA (Hellenic Agricultural Organization “DIMITRA”) is situated. This specific geographic area produces almost 60% of the total honey production in Greece, and the Department of Apiculture is conducting research on different methods of treating *Varroa* with organic acids. Among the used methods is also the one using glycerin strips with oxalic acid and/or formic acid. The aim of the current study was to control the potential influence of veterinary medicines used against *Varroa* mites on the quality of propolis and, moreover, to identify the levels of glycerin, if existent, in propolis samples collected from colonies treated with the organic acids at various times after the treatments, as well as in control colonies receiving no treatment during the experiment’s period. In the present study, five stable beehives were treated with different anti-*Varroa* medicines, while a sixth hive served as an untreated control. Moreover, all colonies had been previously exposed to in-house standard treatment. Three propolis samples (one per sampling date) from each hive (eighteen samples in total) were chemically analyzed through GC-MS, evaluating the impact of each treatment on the chemical composition of propolis, with particular focus on the percentage of glycerol residues in the harvested material. Additionally, Principal Component Analysis (PCA) and its biplot provided insight into the compositional differences resulting from the treatment and sampling time, highlighting variations among chemical groups.

## 2. Materials and Methods

### 2.1. Propolis Samples

Eighteen propolis samples were collected from six bee colonies (A–F) (three per colony). In each colony (A–E), a different treatment of *Varroa destructor* was applied on 27 September 2022, while in F (control samples), no treatment was implemented. All studied samples were collected during autumn-winter of 2022 at days 7, 21, and 65 after the application of anti-*Varroa* treatments in September.

Treatments were applied to five honey bee colonies (A–E) as follows: the A colony received 4 cellulose strips (45 cm × 3 cm × 1.5 mm), which contained dihydrate oxalic acid (Biomus, Lublin, Polland)mixed with glycerin (500 g of oxalic acid/L glycerin; Lazo Medical, Thessaloniki, Greece), the B colony received a 3.5% (*w*/*v*) oxalic acid solution (80 g of dehydrate oxalic acid diluted in 1 Kg of sugar and 1 L of water; all reagents from Lazo Medical, Thessaloniki, Greece), 5 mL of which trickled directly onto bees between comb spaces via a syringe (Chirana 50 ml, Digas, Thessaloniki, Greece). The C colony received 2 plastic strips of amitraz (commercial formulation Apitraz^®^, 500 mg/strip; Zwomelissotexniki, Serres, Greece), D colony received 2.5 g of oxalic acid through sublimation (sublimation temperature 220–230 °C), using a sublimation device (Varroa Pistol basic, Heraklion, Greece). The E colony received 4 cellulose strips (45 cm × 3 cm × 1.5 mm), which contained a mixture consisting of 50% formic acid (99–100%, PanReacAppliChem, Barcelona, Spain), 40% distilled water, and 10% glycerin (Lazo Medical, Thessaloniki, Greece). The control colony (CON) did not receive any treatment during the experiment’s period (Table 1).

In this study, the factors affecting the chemical composition of the collected propolis samples are the date of sampling and the different *Varroa* treatments in each colony, as summarized in Table 1.

### 2.2. Extraction and Sample Derivatization

Propolis samples were extracted extensively with 70% ethanol via dissolution at room temperature for 24 h. Each extract was filtered using filter paper (Whatman, Cytiva, Maidstone, UK) and dried under pressure on a rotary evaporator (Rotavapor R-210, Büchi Labortechnik AG, Flawil, Switzerland) at 40 °C. About 5 mg of each dried extract was silylated with 40 μL of dry pyridine (Sigma-Aldrich, St. Louis, MO, USA) and 60 μL of BSTFA (bis [trimethylsilyl] trifluoracetamide) (Sigma-Aldrich, St. Louis, MO, USA) and heated at 80 °C for 20 min in order to be submitted for GC/MS analysis [3].

### 2.3. GC-MS

The Gas Chromatography–Mass Spectrometry analysis (GC-MS) was conducted using an Agilent Technologies Gas Chromatograph 7820A (Agilent, Santa Clar, CA, USA) connected to an Agilent Technologies 5977B mass spectrometer system with electron impact (EI) ionization at 70 eV. The gas chromatograph is equipped with a split/splitless injector and an HP5MS capillary column (30 m, 0.25 mm internal diameter, and 0.25 μm film thickness). The temperature program used was from 100 to 300 °C at a rate of 5 °C/min. Helium was used as the carrier gas at a flow rate of 0.7 mL/min, with an injection volume of 2 μL, a split ratio of 1:10, and an injector temperature of 280 °C. The identification of the compounds was accomplished by comparing the mass spectra with the Wiley Registry of Mass Spectral Data’s bibliographic data and internal data. The percentage of components in the propolis extract was determined by considering their areas as a percentage of the total ion current.

### 2.4. Principal Component Analysis

The GC-MS data matrix was imported into the multivariate analysis software SIMCA 14.1 (Umetrics, Umeå, Sweden) and analyzed via Principal Component Analysis (PCA), using mean centering with the square root of the standard deviation as a scaling factor (Pareto scaling) and a confidence level set of 95%. Bar charts of the contained metabolites were generated with GraphPad Prism 10 (Boston, MA, USA).

## 3. Results

### GC-MS Analysis

The chemical composition of the studied propolis extracts was investigated via GC-MS analysis after silylation. According to the results, the studied propolis extracts contain 45 identified compounds (Table S1), belonging to the chemical categories of aliphatic acids, aromatic acids and their esters, diterpenes, sesquiterpenes, flavonoids and chalcones, sugars, and glycerol, as shown in Table 2.

The analysis, according to the chemical categories, in all propolis samples, demonstrated the predominance of diterpenes (ranging from 5.21 to 41.87%). The major compounds in the diterpene-rich samples were isocupressic acid, pimaric acid, imbricatoloic acid, totarol, agathadiol, ferruginol, isoagatholal, communic acid, and 13-epi-cupressic acid (Table S1).

**Figure 1:**
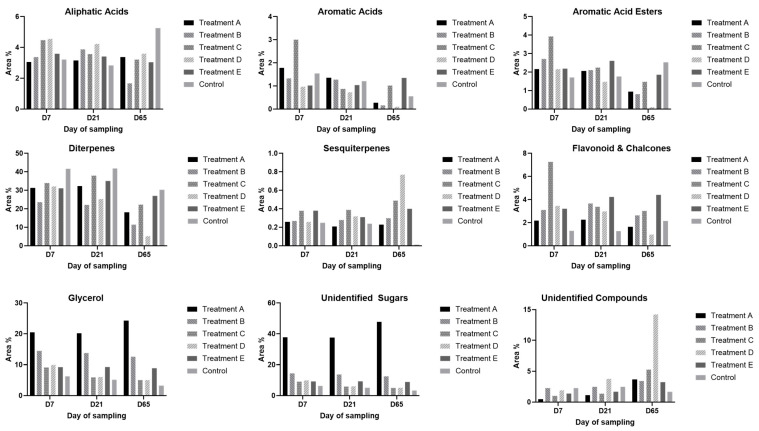
Bar charts of compounds identified via GC-MS for all samples collected on different days (7, 21, and 65 days) after the application of all treatments. A: glycerol-impregnated strips with oxalic acid, B: instillation of oxalic acid, C: sublimation of oxalic acid, D: amitraz strips, E: glycerol-impregnated strips with formic acid, and Control: no treatment.

Among the three different samplings, as shown in Figure 1, it can be observed that in the first two samplings (7 and 21 days after application, respectively) the propolis profile is similar across all colonies. However, in the third sampling (65 days after application), there is either an increased or decreased percentage of glycerol, along with a significant increase in sugars and a noticeable decrease in the percentage of diterpenes.

**Figure 2:**
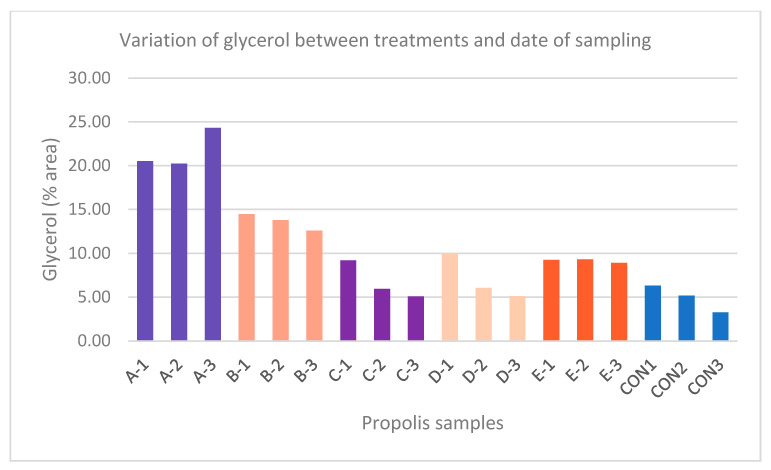
Variation in glycerol percentage between different treatments and dates of sampling. The numbers 1, 2, and 3 indicate the sampling date, which was 7, 21, and 65 days, respectively, from 09/27/2022, when the anti-*Varroa* treatments were applied; the letters A-CON indicate the anti-*Varroa* treatment. A: glycerol-impregnated strips with oxalic acid, B: instillation of oxalic acid, C: sublimation of oxalic acid, D: amitraz strips, E: glycerol-impregnated strips with formic acid, and CON: no treatment = control.

The treatment with amitraz strips (C), the sublimation of oxalic acid (D), and treatment with formic acid (E) with only 10% glycerin had the minimum residues of glycerol of all treatments on the 7th, 21st, and 65th days post treatment (9.19%, 5.95%, and 5.09%; 9.98%, 6.07%, and 5.12%; and 9.25%, 9.30%, and 8.91%, respectively). The control colony had even lower residues of glycerol (6.30%, 5.18%, and 3.27%) (Table 2; Figure 2). However, colony A, the one treated with oxalic acid and glycerin strips, had twice as many residues compared to C, D, and E, while colony B residues were found to be at an intermediate level (Table 2, Figure 2). Interestingly, the glycerol content in colony A increased from 20.51% (A-1) to 24.30% (A-3). For all other treatments, the third sampling had lower glycerol percentages.

**Figure 3:**
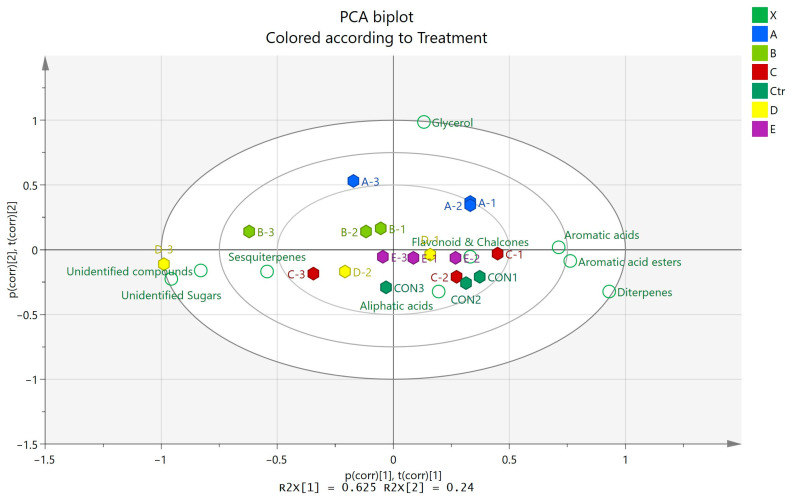
PCA biplot (combined scores and loadings) of propolis samples displaying the relationship between samples and variables (contained metabolites).

PCA was used to examine the intrinsic variation and detect possible outliers, as well as to examine the quality characteristics of the samples. The PCA model was constructed with four principal components (PCs)—R2X (cum) was 0.971 and the Q2 (cum) was 0.464. Additionally, the first two PCs explained 86.4% of the total variance. A PCA biplot (Figure 3) was constructed to facilitate the correlation between different treatments, with the compounds identified via GC-MS analysis. In the first principal component (PC1), a major discrimination was observed for the three different samplings and, in particular, it was observed that the first two samplings (7 and 21 days after application, respectively) tended to group together, while the third sampling (65 days after application) was distinct. For the first two samplings, an increase in aromatic acids, aromatic acid esters, and diterpenes was observed, while sesquiterpenes, unidentified compounds, and sugars predominated in propolis from the third sampling. Control samples (Ctr) were tightly grouped near the center, indicating less metabolic variation, as expected, and the same was observed for samples under treatment E. Samples under treatment C had a greater spread along PC1, with samples collected 65 days after application being closer to sesquiterpenes, while the first two samplings were nearer to flavonoids. In PC2, the second-largest variance was explained; glycerol is far from the center along PC2. Samples under treatment A are in the general direction of glycerol, indicating an association for distinguishing these samples.

## 4. Discussion

The chemical analysis of the propolis samples, as captured via the GC-MS method, revealed compounds, mainly aliphatic acids, diterpenes, sesquiterpenes, aromatic acids, and esters. Additionally, an unexpectedly high percentage of glycerol was found, which is not typically part of the known chemical composition of propolis [3]. Moreover, PCA and the biplot provided a visual summary of how treatments affected samples’ compositions, and which categories of chemical constituents were the most influential in distinguishing between treatments and sampling days. According to de Groot [23], non-polar components detected in propolis, such as fatty acids and their esters, as well as glycerol, are likely to come from beeswax, or from treatments applied to beehives during propolis production. The increased percentage of glycerol observed in the propolis samples in our case could be explained by beekeeping treatments against *Varroa,* involving strips with oxalic acid and glycerol. Due to the efficacy of this method, the utilization of such strips is widespread, which could explain the high percentage of glycerol residues in general, even in control colonies, due to their previous exposure to such treatments. Although this beekeeping practice has no side effects for bees and colonies and does not affect, according to the literature, honey and wax [21], the changes in the chemical composition of propolis should be highlighted [1].

In colonies A and E, where glycerol-impregnated oxalic acid strips and formic acid strips, respectively, were applied, the glycerol percentages in samples A-1 (20.51%) and E-1 (9.25%) differed, due to the fact that the glycerol content in oxalic acid strips is higher (500 g/L glycerol) than in formic acid strips (50% formic acid; 40% water; 10% glycerin).

The high glycerol residues obtained from treatments with the strips of oxalic acid impregnated with glycerin could negatively impact the quality of the harvested propolis for further applications. High glycerol concentrations could adversely affect the organoleptic characteristics of food products containing propolis, due to its hygroscopic, sticky nature and sweet taste, making the products less desirable for consumption. Additionally, it may have side effects on the human body, such as a laxative effect [24].

Our results showed that the levels of glycerol were reduced, without being eliminated, after the 65 days of treatment without glycerin. The lack of complete elimination may be due to the use of glycerin-containing products on all colonies prior to this study [3]. It is also important to note that although the amitraz-treated colony showed a low amount of glycerol, amitraz is a synthetic formamidine acaricide, and its use can lead to residue accumulation in hive products, as well as the development of resistant *Varroa* populations, raising concerns about its long-term safety and sustainability. Rodríguez et al. [25] have shown that mites have developed increased resistance to it, and that it is also associated with alterations in honey bee physiology [26,27,28,29]. Unlike amitraz, the sublimation or instillation of oxalic acid inside the hive has been shown to not negatively affect bee colony fitness or successful wintering [30].

It is suggested that the application of glycerin strips in the case of oxalic acid (A), as can be seen from Figure 2, caused a high percentage of glycerol from the first sampling (20.51%). High levels were maintained in the second sampling (20.23%), while the percentage increased in the third (24.30%).

Applying glycerin strips with formic acid (E) resulted in a much lower glycerol percentage than in the previous practice, decreasing from 9.25% to 8.91%, although this was a small change considering that 65 days had passed since the treatments were initially implemented.

In the control samples from bee colony F, the percentage of glycerol residue decreased over time, which can be attributed to the transport of glycerol outside the hive, either due to its adhesion to the bees’ bodies [31] or its degradation by microorganisms that naturally use it as a carbon and energy source [32].

The chemical analysis of all propolis samples, with or without anti-*Varroa* treatments in hives, mainly showed the existence of diterpenes. This rich diterpenic profile could be attributed to the existence of coniferous trees, plants of the Cupressaceae and Pinaceae families, which are widespread in northeast Greece [33]. This characteristic profile of Mediterranean propolis, with its special biological activities [3], could be compromised when glycerol is present at such high percentages.

Τhe fact that a similar propolis profile was observed in the first two samplings from all hives can be attributed to the stationary beekeeping applied throughout the experiment and sampling being conducted within the same season (autumn). However, the third collection took place in the winter, and a different chemical profile was observed, with an increased percentage of sugars and a simultaneous decrease in diterpenes. Autumn is the period when bees collect the largest amount of resin [34], while in winter, bees are inside the hive, and their breeding is carried out with food stocks and supplementary sweeteners from the beekeepers.

## 5. Conclusions

In conclusion, sublimation or the instillation of oxalic acid preserves the quality of propolis, while oxalic acid glycerin strips alter its composition and reduce its quality. Formic acid glycerin strips may have a lesser negative impact on propolis, but their efficacy for *Varroa* control requires further investigation. These findings emphasize that beekeeping practices directly influence the chemical composition and quality of propolis. Based on our results, the recommendations could be forwarded to national authorities and beekeepers’ associations to avoid anti-*Varroa* treatments using oxalic acid and glycerol-impregnated strips when propolis is planned to be harvested for medicinal, nutritional, or cosmeceutical applications.

## Figures and Tables

**Table 1:** Propolis samples with anti-*Varroa* treatments.

Bee Colonies	Propolis Samples	Anti-*Varroa* Treatments
Colony A	A-1/A-2/A-3	4 strips with oxalic acid and glycerin (500 g/L of glycerin)
Colony Β	Β-1/Β-2/Β-3	Instillation of oxalic acid (80 g of oxalic acid in a 1:1 solution of sugar–water)
Colony C	C-1/C-2/C-3	2 strips with amitraz (Apitraz^®^ 500 mg/strip)
Colony D	D-1/D-2/D-3	Sublimation of oxalic acid (2.5 g/colony)
Colony E	E-1/E-2/E-3	4 strips with formic acid, water, and glycerin (50:40:10)
Colony F	CON1/CON2/CON3	No treatment

**Table 2:** Categorization of components detected via GC-MS in propolis samples.

	Area %																	
Chemical Category	A-1	A-2	A-3	B-1	B-2	B-3	C-1	C-2	C-3	D-1	D-2	D-3	E-1	E-2	E-3	CON1	CON2	CON3
Aliphatic acids	3.05	3.15	3.38	3.38	3.88	1.67	4.48	3.57	3.22	4.56	4.23	3.60	3.59	3.41	3.04	3.22	2.84	5.25
Aromatic acids	1.78	1.36	0.27	1.33	1.28	0.16	3.01	0.88	1.02	0.97	0.73	<0.1	1.02	1.04	1.35	1.54	1.21	0.56
Aromatic acid esters	2.17	2.06	0.95	2.72	2.11	0.81	3.94	2.25	1.48	2.16	1.48	<0.1	2.19	2.61	1.86	1.72	1.77	2.53
Diterpenes	31.41	32.27	18.07	23.59	22.16	11.50	34.04	38.02	22.32	32.13	25.34	5.21	31.09	35.12	27.00	41.65	41.87	30.24
Sesquiterpenes	0.26	0.21	0.23	0.27	0.28	0.30	0.38	0.39	0.49	0.26	0.32	0.77	0.38	0.31	0.40	0.25	0.24	-
Flavonoid and Chalcones	2.19	2.25	1.66	3.10	3.66	2.64	7.27	3.39	3.02	3.46	2.99	0.98	3.21	4.23	4.41	1.30	1.26	2.16
Glycerol	20.51	20.23	24.30	14.48	13.78	12.60	9.19	5.95	5.09	9.98	6.07	5.12	9.25	9.30	8.91	6.30	5.18	3.27
Unidentified Sugars	37.72	37.63	47.70	49.23	50.59	63.89	36.19	44.41	58.36	45.17	55.51	66.39	48.21	42.69	50.06	41.28	44.10	52.86
Unidentified compounds	0.51	1.16	3.69	2.28	2.51	3.46	1.03	1.39	5.29	1.92	3.80	14.22	1.41	1.70	3.26	2.30	2.47	1.69

## Data Availability

The original contributions presented in this study are included in the article/Supplementary Material. Further inquiries can be directed to the corresponding author.

## Supplementary Materials

The following supporting information can be downloaded at: https://www.mdpi.com/article/10.3390/insects16090871/s1. Table S1: Chemical composition (% area) from GC-MS analysis of Greek propolis samples with and without anti-*Varroa* treatments (A1–E3 and CON1–CON3).
