# Supplementary material for: Does Glycerin Used in Varroa Treatments Alter Propolis Quality?
Source: Insects. 2025 Aug 22;16(9):871. doi: 10.3390/insects16090871 (PMC12470576; doi:10.3390/insects16090871)
Supplement: Supplementary file 1 [file insects-16-00871-s001.zip › insects-3803630-supplementary.pdf]

Supplementary Materials

Table S1. Chemical composition (% area) by GC-MS analysis of Greek propolis samples with and without anti-*Varroa* treatments (A1-E3 and CON1-CON3).

		Area %																	
**RT (min)**	**COMPOUND**	**A-1***	**A-2***	**A-3***	**B-1***	**B-2***	**B-3***	**C-1***	**C-2***	**C-3***	**D-1***	**D-2***	**D-3***	**E-1***	**E-2***	**E-3***	**CON1 ***	**CON2 ***	**CON3 ***
6.10	glycerol	20.51	20.23	24.30	14.48	13.78	12.60	9.19	5.95	5.09	9.98	6.07	5.12	9.25	9.30	8.91	6.30	5.18	3.27
6.67	succinic acid																		0.38
10.56	malic acid	0.56	0.26	0.54	0.43	0.55	0.46	0.65	0.14	0.14	0.25		0.83	0.22	0.14		0.22	0.20	1.41
11.28	cinnamic acid				0.18	0.16										0.22			
12.41	α-cedrol	0.26	0.21	0.23	0.27	0.28	0.30	0.38	0.39	0.49	0.26	0.32	0.77	0.38	0.31	0.40	0.25	0.24	
13.42-14.36	sugars	0.65	0.69	0.96	0.58	0.99	1.54	0.14	0.74	1.38	0.65	1.65	3.54	0.67	0.77	1.29	0.32	0.25	0.54
15.35	pentanedioic acid	0.10	0.11	0.09	0.14	0.17	0.23	0.07	0.14	0.14	0.21	0.16	0.43	0.16	0.17	0.12	0.08	0.08	0.16
15.58-21.17	sugars	35.45	34.84	45.71	46.08	46.65	58.50	33.90	40.12	53.46	41.88	50.54	60.13	44.35	38.91	44.86	38.73	40.63	50.16
20.26	gallic acid																0.85	0.51	
21.02	3,4 dimethoxy cinnamic acid	0.38						0.75											
21.39	manool	0.59	0.59					<0.1			<0.1								
21.46	palmitic acid	0.95	1.01	1.46	1.68	1.88	1,92	2.15	1.99	1.97	2.49	2.59	1.97	2.10	2.02	2.01	1.88	1.51	2.09
21.63 - 21.99	sugars	0.38	0.38	0.27	0.50	0.63	1.15	0.14	0.85	1.12	0.67	1.22	1.70	0.68	0.60	0.83	0.25	0.42	0.70
22.06	ferulic acid	0.14	0.15		0.21	0.19		0.50	0.17	0.24	0.18			0.18	0.15	0.17	0.22	0.24	0.31
22.31	isoferulic acid	0.16	0.14		0.24	0.22	0.16	0.37	0.15		0.18	0.19		0.19	0.21	0.22			
23.03	sugar	0.23	0.20	0.15	0.24	0.25	0.33	0.32	0.24	0.25	0.29	0.28	0.21	0.32	0.28	0.28			0.43
23.25	caffeic acid	0.51	0.48	0.27	0.70	0.71		1.39	0.56	0.78	0.61	0.54		0.65	0.68	0.74	0.47	0.46	0.26
24.03	sugar	0.06			0.15	0.38	0.19		0.12					0.12	0.07	0.08			
24.10	pentenyl p-coumarate	0.28	0.21	0.20	0.12				0.25		0.20			0.19	0.24		0.21	0.22	0.23

24.31	linoleic acid	0.36	0.40	0.23	0.26	0.34	0.32	0.40	0.40	0.27	0.40	0.39		0.36	0.32	0.31	0.36	0.36	0.29
24.44	oleic acid	1.08	1.37	1.06	0.87	0.94	0.66	1.21	0.90	0.70	1.21	1.09	0.37	0.75	0.76	0.60	0.69	0.70	0.94
24.71	sempervirol	0.34	0.34	0.20	0.27	0.25	0.13	0.41	0.40	0.26	0.36	0.32		0.32	0.39	0.26	0.37	0.37	0.40
24.98	feruginol	2.42	2.35	1.18	1.73	1.54	0.77	2.30	2.18	1.27	2.29	1.68	0.49	2.21	2.40	1.88	2.78	2.64	1.79
25.16	14,15-dinor-13-oxo-8(17)-labden-19-oic acid	0.40	0.40	0.23	0.37	0.34	0.21	0.48	0.36	0.28	0.61	0.44		0.38	0.39	0.29			
25.37	sugar	0.20	0.18		0.35	0.17													
25.49	copalol	0.53	0.62	0.32	0.37	0.31	0.17	0.53	0.57	0.33	0.60	0.44		0.47	0.58	0.34	0.91	0.93	0.49
25.7	communic acid	0.54	0.53	0.30	0.59	0.49	0.25	0.72	1.31	0.70	0.67	0.46		0.52	0.57	0.39	0.44	0.41	3.68
25.99	pentenyl ferulate	0.45	0.48	0.22	0.41	0.41	0.17	0.74	0.72	0.44	0.51	0.40		0.45	0.54	0.38	0.46	0.49	
26.01	diterpenic acid																		1.75
26.17	pimaric acid	3.25	2.98	1.87	2.41	2.45	1.23	3.64	5.69	2.53	3.72	2.52	0.57	3.42	4.49	2.96	3.48	3.46	5.79
26.28	totarol	4.21	4.25	2.14	2.98	3.30	1.56	5.10	4.59	2.91	4.25	3.59	0.78	4.08	4.70	3.14	5.37	7.03	1.73
26.72	4-pentenyl ester of trans-isoferulic acid	0.23	0.22		0.29	0.25		0.43	0.26		0.21			0.25	0.38	0.21	0.22	0.20	0.50
26.83	3-methyl-3-butenyl ester of caffeic acid																		0.71
26.96	pimaric acid & imbricataloic acid	6.67	6.58	3.53	5.31	4.99	2.88	7.83	6.99	4.84	6.54	5.23	1.21	6.67	7.94	6.72	7.39	6.66	1.63
27.07	dehydroabietic acid	0.21	0.20	0.18	0.15	0.18		0.32	0.17		0.20	0.35		0.16	0.17	0.14	0.18	0.16	0.17
27.34	13-epi-torulosol	0.11	0.12	0.13					0.16					0.17	0.16		0.19	0.20	
27.51	2 methyl-2-butenyl ester of caffeic acid	0.52	0.42	0.31	0.73	0.63	0.32	0.98	0.46	0.36	0.51	0.70		0.61	0.68	0.58	0.39	0.41	0.28
27.67	3-methyl-2-butenyl ester of caffeic acid	0.38	0.39	0.22	0.69	0.50	0.32	1.07	0.45	0.68	0.44	0.38		0.56	0.65	0.57	0.26	0.27	0.62
27.94	13-epi-cupressic acid	0.48	0.59	0.67	0.32	0.39	0.19	0.56	1.09	0.49	0.56	0.62		0.97	0.82	0.69	1.23	1.14	0.46

28.16	totarolon	0.30	0.25	0.15	0.22	0.26	0.10	0.28	0.27		0.22	0.16		0.25	0.28	0.18	0.25	0.25	0.17
28.7	isoagatholal	1.72	1.87	0.90	1.13	0.93	0.43	1.63	1.50	0.66	1.61	1.12	0.19	1.49	1.49	1.13	2.90	2.96	0.90
28.87	pinostrobin chalcone	0.46	0.38	0.27	0.72	0.82	0.33	1.32	0.61	0.34	0.56	0.47		0.68	1.01	0.85	0.32	0.31	
29.26	totarolon derivative	0.15	0.15						0.22		0.18			0.15			0.12	0.12	0.46
29.52	pinocembrin chalcone	0.63	0.59	0.41	0.84	1.09	0.51	2.34	1.05	0.84	0.92	0.92	0.33	0.95	1.38	1.37	0.63	0.61	0.77
29.69	agathadiol & imbricatoloic acid	2.51	2.91	1.62	1.98	1.67	0.88	2.58	4.90	3.43	2.77	2.39	0.67	2.26	2.20	1.83	3.87	3.92	9.45
30.38	isocupressic acid	7.36	7.88	4.49	5.53	4.88	2.70	7.36	7.37	4.62	7.26	5.84	1.30	7.34	8.31	6.89	11.85	11.27	2.83
30.46	pinobanksin	0.68	0.77	0.66	1.00	1.20	1.40	1.52	1.07	1.38	1.16	1.16	0.65	1.02	1.18	1.70			1.02
30.92	junicedric acid	0.21	0.25	0.16	0.23	0.18		0.30	0.25		0.29	0.18		0.23	0.23	0.16	0.34	0.37	0.32
31.35	sugar	0.41	0.52	0.38	0.70	0.70	0.85	1.03	0.70	0.81	0.70	0.58	0.32	0.69	0.78	0.94	0.87	1.16	0.55
31.60	pinobanksin 3-O-acetate	0.23	0.30	0.21	0.35	0.32	0.27	1.10	0.40	0.27	0.51	0.26		0.33	0.39	0.27	0.19	0.19	0.20
31.86	sugar	0.16	0.43	0.23	0.20	0.20	0.21	0.19	0.18		0.21	0.18		0.18	0.16	0.14	0.20	0.19	0.16
32.07	benzyl ester of caffeic acid	0.18	0.24		0.29	0.20		0.43			0.17						0.11	0.10	0.19
32.14-32.27	sugars	0.18	0.39		0.43	0.62	1.12	0.47	1.46	1.34	0.77	1.06	0.49	1.20	1.12	1.64	0.91	1.47	0.34
32.65	pinobanksin derivative							0.16											
32.73	galangin	0.19	0.21	0.11	0.19	0.23	0.13	0.83	0.26	0.19	0.31	0.18		0.23	0.27	0.22	0.17	0.16	0.18
33.20	phenylethyl ester of caffeic acid	0.13	0.10		0.19	0.12		0.29	0.11		0.12			0.13	0.12	0.12	0.09	0.09	

*1, 2, 3: propolis sampling after 7, 21, 65 days of the initial implementation of the practices, respectively. A: oxalic acid strips with glycerol, B: oxalic acid instillation, C: amitraz strips, D: oxalic acid sublimation, E: formic acid strips with glycerol, CON: no treatment
